# Improvement in the Detection of Cystic Metastatic Papillary Thyroid Carcinoma by Measurement of Thyroglobulin in Aspirated Fluid

**DOI:** 10.1155/2016/8905916

**Published:** 2016-01-04

**Authors:** Yong Wang, Huan Zhao, Yi-Xiang J. Wang, Min-Jie Wang, Zhi-Hui Zhang, Li Zhang, Bin Zhang, Anil T. Ahuja, Chun-Wu Zhou, Yu-Xin Jiang, Hui-Qin Guo

**Affiliations:** ^1^Department of Diagnostic Ultrasound, Cancer Hospital & Institute, Peking Union Medical College and Chinese Academy of Medical Sciences, Beijing 100021, China; ^2^Department of Pathology, Cancer Hospital & Institute, Peking Union Medical College and Chinese Academy of Medical Sciences, Beijing 100021, China; ^3^Department of Imaging and Interventional Radiology, Prince of Wales Hospital, The Chinese University of Hong Kong, Shatin, New Territories, Hong Kong; ^4^Department of Clinical Library, Cancer Hospital & Institute, Peking Union Medical College and Chinese Academy of Medical Sciences, Beijing 100021, China; ^5^Department of Cancer Epidemiology, Cancer Hospital & Institute, Peking Union Medical College and Chinese Academy of Medical Sciences, Beijing 100021, China; ^6^Department of Head & Neck Surgery, Cancer Hospital & Institute, Peking Union Medical College and Chinese Academy of Medical Sciences, Beijing 100021, China; ^7^Department of Diagnostic Imaging, Cancer Hospital & Institute, Peking Union Medical College Hospital, Peking Union Medical College and Chinese Academy of Medical Sciences, Beijing 100021, China; ^8^Department of Diagnostic Ultrasound, Peking Union Medical College Hospital, Peking Union Medical College and Chinese Academy of Medical Sciences, Beijing 100730, China

## Abstract

Cystic change in metastatic lymph nodes of papillary thyroid carcinoma (PTC) is a diagnostic challenge for fine needle aspiration (FNA) because of the scant cellularity. The aim of this study was to evaluate the measurement of thyroglobulin in fine needle aspirate (Tg-FNA) for detecting metastatic PTC in patients with cystic neck lesions and to validate the optimal cutoff value of Tg-FNA. A total of 75 FNA specimens of cystic lesions were identified, including 40 of metastatic PTC. Predetermined threshold levels of 0.04 (minimum detection level), 0.9, 10.0, and 77.0 ng/mL (maximum normal serum-Tg level) were used to evaluate the diagnostic accuracy of Tg-FNA for metastatic PTC detection. The areas under the receiver operating characteristic curve for diagnosing metastatic PTC of Tg-FNA values of 0.04, 0.9, 10.0, and 77.0 ng/mL were 0.5 (95% confidence interval [CI], 0.382–0.618), 0.645 (95% CI, 0.526–0.752), 0.945 (95% CI, 0.866–0.984), and 0.973 (95% CI, 0.907–0.996), respectively. With a cutoff value of 77.0 ng/mL, the combination of Tg-FNA and FNA cytology showed superior diagnostic power (97.5% sensitivity and 100% specificity) compared to FNA cytology alone (80% sensitivity and 100% specificity). We recommend a Tg-FNA cutoff of 77.0 ng/mL, the maximum normal serum-Tg level, for cystic neck lesions.

## 1. Introduction

Fine needle aspiration cytology (FNAC) is the most important modality for the evaluation of lymphadenopathy. It is highly specific and sensitive in patients with solid lesions [1]. However, for cystic lesions, the lack of epithelium in cyst aspiration may lead to a false negative interpretation of FNAC results [2, 3]. Cystic lymph node metastasis has been detected in 5.2% of malignant aspirates, and the tumor type that most frequently causes cystic change is papillary thyroid carcinoma (PTC) [4]. An ultrasonographic cystic appearance of cervical lymph nodes has been observed in 70% of metastatic PTC cases [5].

The measurement of thyroglobulin in fine needle aspirate (Tg-FNA) was initially proposed by Pacini et al. in 1992 for the detection of neck lymph node metastases in patients with PTC [6]. Several studies have reported that Tg-FNA is more sensitive than FNAC for detecting metastatic PTC and that the sensitivity of FNAC is increased when combined with Tg-FNA [3, 7–15]. However, only a few studies with a limited number of cases have focused on the utility of Tg-FNA for cystic lesions [3, 12]. The diagnostic threshold has not been well established. The wide range of suggested cutoff values for Tg-FNA in previous studies might be due to the differences in study populations. Some studies included only patients with PTC who had undergone surgery and radioiodine ablation [9–12], while others included those awaiting thyroid surgeries [13]. In addition, most studies examined Tg-FNA in selected lymph nodes, histologically proved to be either metastatic PTC or reactive hyperplasia [7–15]. The diagnostic threshold was determined based on strictly selected study populations, which may limit the general use of Tg-FNA in clinical practice.

In this study, we evaluated the value of Tg-FNA in 75 cystic lesions, the largest reported series of cystic lesions to date, and primarily examined this technique in a wider clinical context, including metastatic PTC in patients who had undergone or were yet to undergo surgery, lymph node metastases from extrathyroidal malignancies, and lesions of cervical origin. The aim of this study was to evaluate the use of Tg-FNA for detecting metastatic PTC in patients with cystic neck lesions and to validate the optimal cutoff value of Tg-FNA.

## 2. Materials and Methods

### 2.1. Case Selection

The specimens were consecutively collected from the Cancer Institute/Hospital, Chinese Academy of Medical Sciences (CAMS), between September 2012 and December 2014. Patients were selected on the basis of the following criteria: (1) having cystic neck lesions and being referred to FNA examination by their physicians and (2) the fluid aspirated from the cervical lesion being nonpurulent. FNAC and Tg-FNA measurement were performed on the enrolled patients. All patients provided informed consent before FNA. This study protocol was reviewed and approved by the ethics committee of the Cancer Institute/Hospital, CAMS.

### 2.2. FNAC

Palpable lesions were aspirated by cytopathologists, and nonpalpable lesions were aspirated by experienced radiologists under the real-time ultrasound guidance. FNA was performed using a 22-gauge needle attached to 10 mL syringe without the aid of a syringe holder. Several drops of aspirated fluid were first added to 0.5 mL of normal saline solution for Tg-FNA. The residual fluid in the needle was then rinsed in CytoLyt (Hologic, Marlborough, MA, USA) to prepare a ThinPrep (Hologic) slide. Slides were fixed in alcohol and stained with Papanicolaou staining. They were then interpreted by cytologists with experience ranging from 5 to 18 years. All cases, except those diagnosed cytologically as benign, were reviewed in the daily conference among these cytopathologists.

### 2.3. Tg-FNA

Specimens were stored at −20°C and transferred to the clinical laboratory for thyroglobulin analysis in one month. Tg concentrations were measured using an automated electrochemiluminescence immunoassay (Cobas e 601, Roche Diagnostics, Manheim, Germany). The minimum detectable Tg concentration was 0.04 ng/mL.

### 2.4. Data Analysis and Statistical Analysis

Positive final diagnoses were based on histological confirmation of metastatic PTC or cytological diagnosis of PTC. Negative final diagnoses were made for lymph nodes free of metastatic disease based on cytology and negative follow-up imaging findings for at least 12 months, histologically or cytologically confirmed lymph node metastases from extrathyroidal malignancies, and histologically or cytologically confirmed benign lesions of cervical origin.

Cytology results were grouped into two categories according to the cytology report. Cases with reports documenting metastases from PTC and those documenting suspicious metastases from PTC were considered positive. Negative diagnoses were assigned to (1) cases with reports where “atypical” was mentioned but “metastasis” was not, (2) cases of lymph node metastases from extrathyroidal malignancies, (3) cases of lymph nodes with reactive hyperplasia, and (4) cases with specific benign findings.

To interpret Tg-FNA, four threshold levels were decided according to previous reports [7–16]: 0.04 ng/mL (the Tg detection limit according to the manufacturer's instructions in our study), 0.9 ng/mL (the Tg detection limit according to previous studies), 10.0 ng/mL, and 77.0 ng/mL (the maximum level of normal serum-Tg according to the manufacturer's instructions and proved by evaluation of 100 healthy controls). Tg-FNA was considered positive or negative for values higher or lower than the threshold values, respectively.

Receiver operating characteristic (ROC) curve analysis was conducted to determine the most appropriate threshold value for Tg-FNA, with the areas under the ROC curve (AUC) and confidence intervals (CI) being assessed with MedCalc Version 14.10.2. Tg-FNA median values were compared using the Kruskal-Wallis test. The two-sided alpha error level of 0.05 was adjusted to 0.05/N using the Bonferroni correction for multiple comparisons. Statistical analyses were performed with SPSS 12.0 (SPSS Inc., Chicago, IL, USA).

## 3. Results

In total, 75 cystic aspirates were included in our study. The cervical cystic diseases were as follows: (1) 40 cases of metastatic PTC (39 diagnosed as pure metastatic PTC and one with a major squamous cell carcinoma component and a minor papillary carcinoma component, surgically confirmed to be PTC recurrence), 5 cases of reactive lymphadenitis (all in patients with a history of thyroidectomy for PTC), 15 cases of lymph node invasion from metastatic extrathyroidal malignancies (11 squamous carcinomas and 4 adenocarcinomas), and 15 cases of benign lesions of cervical origin (2 branchial cleft cysts, 3 thyroglossal cysts, 4 lymphangiomas, 2 schwannomas, and 4 cystic lesions of the salivary glands) (Table 1).

Table 1 also shows the Tg-FNA values in the different groups of cystic lesions. Cystic metastases from PTC (median, >500 ng/mL) showed significantly higher levels on Tg-FNA than reactive lymphadenitis (median, 4.43 ng/mL, *P* < 0.001), metastases from extrathyroidal cancer (median, 1.38 ng/mL, <0.001), and benign lesions of cervical origin (median, 2.04 ng/mL, *P* < 0.001). There were two special cases. One was of a metastatic PTC, which had a very low value on Tg-FNA (0.64 ng/mL). This case was diagnosed histologically as metastatic thyroid carcinoma with the tumor mainly composed of squamous carcinoma and a small component of papillary carcinoma. The other case was of a thyroglossal cyst, which showed a Tg-FNA level as high as that for metastatic PTC (>500 ng/mL).

**Figure 1:**
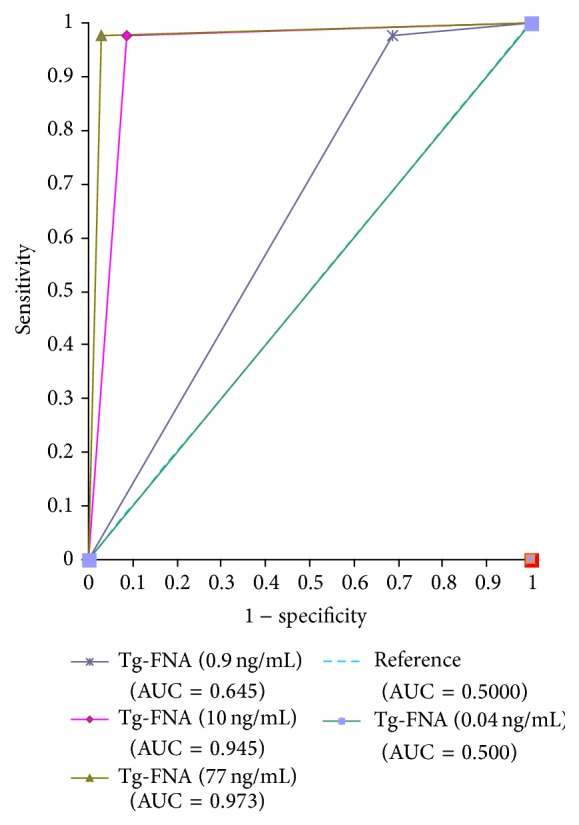
ROC curves for Tg-FNA for different cutoff values.

Based on the final diagnosis, 40 lesions were metastatic PTCs and the remaining 35 were not. We evaluated the optimal cutoff value for Tg-FNA in diagnosing metastatic PTC, and four threshold values were used: 0.04, 0.9, 10.0, and 77.0 ng/mL. The AUCs for determining metastatic PTC of Tg-FNA levels of 0.04, 0.9, 10.0, and 77.0 ng/mL were 0.5 (95% CI, 0.382–0.618), 0.645 (95% CI, 0.526–0.752), 0.945 (95% CI, 0.866–0.984), and 0.973 (95% CI, 0.907–0.996), respectively. The AUC of the Tg-FNA cutoff of 77.0 ng/mL was the highest, significantly higher than the AUCs of the Tg-FNA cutoffs of 0.04 ng/mL (*P* < 0.001) and 0.9 ng/mL (*P* < 0.001), but not significantly higher than the AUC of the Tg-FNA cutoff of 10.0 ng/mL (*P* = 0.157) (Figure 1).

A diagnostic strategy of FNAC alone had a sensitivity of 80.0% and a specificity of 100% for determining metastatic PTC. A higher sensitivity (97.5% versus 80%, *P* = 0.013) and similar specificity (100% versus 100%, *P* = 1.000) were obtained for a diagnostic strategy of Tg-FNA combined with FNAC, compared to FNAC alone. In this strategy, a positive result was considered if the criteria for either test were met, except in one special case. In this case, the Tg level was positive (>500 ng/mL), but the definitive cytological diagnosis was thyroglossal cyst. Accordingly, a diagnosis of thyroglossal cyst with a high Tg level was a reasonable diagnosis. This case was considered negative when performing statistical analyses of the combined criteria (Table 2).

## 4. Discussion

Aided with FNAC, ultrasound technique is the main modality for assessing thyroid gland lesions [17–19]. A cystic appearance of lymph nodes is a characteristic of metastatic PTC [2, 3, 20, 21]. However, frequent nondiagnostic cytologic results and high false negative rates for FNA are reported in cases of cystic metastatic lymph nodes [2, 3]. In the past, we recommended thyroid examination in cases of inadequate cells in a cervical cystic aspirate, especially when the patient had a history of PTC or the fluid aspirated was brown in color. However, the results were not always as expected. It should be considered that many benign lesions or extrathyroidal malignancies also have a cystic appearance, as shown in Table 1. Of the 75 cystic cervical lesions included in our study, only 40 were proven to be metastatic PTC. Therefore, an objective examination for the detection of PTC in cystic lesions was needed.

Tg-FNA was initially proposed by Pacini et al. in 1992 and has been reported to increase the detection rate of metastatic PTC combined with FNAC [3, 7–16]. However, the diagnostic threshold has not been well established. In the present study, we suggested four threshold values, as in previous reports [7–15]. The functional sensitivity of Tg measurement was the most commonly used threshold values in previous studies [7, 10–12, 16]. However, the use of this threshold value is associated with several limitations. If the aspirates are contaminated with blood containing high levels of Tg, the Tg-FNA values may be higher than the set threshold value, even if the lymph nodes are not metastatic. In patients awaiting surgery and even in patients who have undergone surgery without radioiodine ablation, serum-Tg may not be suppressed, which can confuse the diagnosis. This hypothesis was proven in our study. In the present study, Tg levels from all lymph node (both benign and malignant) aspirates were above the detectable level, which may partly be due to the high sensitivity of our detection method. According to the manufacturer's instructions, the minimum detectable Tg concentration is 0.04 ng/mL, indicating a higher sensitivity than in previous studies [7, 10–12, 16]. When 0.9 ng/mL, the reported detectable Tg level in previous studies [7, 10, 16], was used as the threshold, the AUC was only 0.645, indicating poor diagnostic ability.

The serum-Tg level has also been used as the threshold value [14, 22, 23]. A Tg-FNA/serum-Tg ratio of >1.0 is interpreted as a positive result. However, this method could not be used for patients who had not undergone serum-Tg evaluation. In addition, in cases where blood sampling for serum-Tg and Tg-FNA was not performed simultaneously but within a few days to a few weeks, hormonal variation in serum-Tg could skew the data. Therefore, we used the maximum normal serum-Tg level (77.0 ng/mL) as the threshold value, which was found to be the most valuable threshold value in our study. Another previously reported threshold value (10.0 ng/mL) was also found to be of value [14, 24]. The specificity of 10.0 ng/mL was lower than that of 77.0 ng/mL. The Tg-FNA level was higher than 10.0 ng/mL in one case of lymph node metastasis from an extrathyroidal malignancy (21.12 ng/mL) and in one case of schwannoma (50.20 ng/mL). However, none of the tested threshold values could distinguish benign from metastatic lymph nodes in patients with PTC with complete reliability. The cutoff value of 77.0 ng/mL (the normal serum-Tg level) was found to have the best diagnostic performance.

Of the cystic metastatic PTC cases, 20% (8 of 40 cases) yielded a cytologically equivocal or nondiagnostic result. This is similar to the rate reported in previous studies [7, 12] and reflects the challenge in diagnosing cystic cervical lesions with FNAC. The sensitivity for the detection of lymph node metastasis was increased greatly by combining Tg-FNA and cytology; 7 of 8 metastatic lymph nodes with negative cytology were detected using Tg-FNA. One case of metastatic PTC missed by Tg-FNA was diagnosed histologically as metastatic thyroid carcinoma, where the tumor was mainly composed of squamous carcinoma with a small component of papillary carcinoma. This phenomenon has been reported by Boi et al. [25]. In their series, 2 of the 4 metastatic lymph nodes undetectable by Tg-FNA were from anaplastic thyroid tumors, and the other 2 were from very undifferentiated PTC. Tg will not be detectable not only in lymph nodes with metastasis from anaplastic or undifferentiated PTC, but also in lymph nodes with metastasis from recurrent PTC. Because 2% to 5% of differentiated thyroid carcinomas are reported to lose their differentiated features, making monitoring by serum-Tg difficult, recurrent non-RAI-avid 18F-fluorodeoxyglucose-positron emission tomography-positive disease has been reported to develop, leading to the patient's death [26]. A similar case was encountered in our study. The patient had a history of thyroidectomy for PTC with three recurrences. Thus, we recommend combined cytology and Tg-FNA rather than either technique alone to detect any histological type of thyroid cancer metastases [25].

## 5. Conclusion

The results of the current study demonstrate that Tg measurement in FNA material appears to be a useful ancillary test that improves the detection of cystic PTC metastases, and the maximum normal serum-Tg level (77.0 ng/mL) is suggested as the threshold value with a good diagnostic performance for cystic lesions.

## Figures and Tables

**Table 1:** Correlations between the final diagnosis and the Tg-FNA values.

Final diagnosis	Case number	Tg-FNA values (ng/mL) range/median
Metastatic PTCs^a^	40	0.64–500+ 500+
Reactive lymphadenitis^b^	5	2.10–5.74 4.43
Metastases from extrathyroidal malignancies	15	0.10–21.15 1.38
Cervical original benign lesions	15	0.09–500+ 2.04
Branchial cleft cysts	2	0.09–0.12 0.10
Thyroglossal cysts	3	5.40–500+ 6.22
Lymphangiomas	4	0.35–3.20 1.48
Schwannomas	2	2.04–50.20 26.12
Cystic lesions of the salivary gland	4	0.10–2.54 1.29

^a^One case of metastatic PTC was diagnosed histologically as metastatic thyroid carcinoma where the tumor was mainly composed of squamous carcinoma with a small component of papillary carcinoma. The Tg-FNA value of this case was 0.64 ng/mL and was the lowest of the metastatic PTCs.

^b^All 5 cases had a history of thyroidectomy for thyroid papillary carcinoma. These negative lymph nodes were followed up by sonography for at least 12 months and showed a decrease in size.

**Table 2:** Evaluation of metastatic PTC according to the diagnostic modality.

Modalities	SN	SP	PPV	NPV	AC
FNAC	80.0%	100%	100%	81.4%	89.3%
Tg-FNA^a^	97.5%	97.1%	97.5%	97.1%	97.3%
FNAC + Tg-FNA^b^	97.5%	100%	100%	97.1%	98.7%

^a^Tg-FNA refers to the optimal cutoff value of Tg-FNA at 77.0 ng/mL.

^b^Positive result was determined if the positive criteria for either criterion were met except in one special case. In this case, the Tg level was positive (>500 ng/mL), but the definitive cytological diagnosis was thyroglossal cyst. Accordingly, a diagnosis of thyroglossal cyst with a high Tg level was a reasonable diagnosis. This case was considered negative when performing statistical analyses of the combined criteria. SN, sensitivity; SP, specificity; PPV, positive predictive value; NPV, negative predictive value; AC, accuracy.

## Study Limitations

There are some limitations to our study. Five (100%) reactive lymph nodes, 7 (46.7%) metastases from extrathyroidal malignancies, and 7 (46.7%) benign lesions of cervical origin were not surgically confirmed. However, all of these nonmetastatic PTC lesions had definite results on FNA and the Tg-FNA level was <77.0 ng/mL. Moreover, the 5 reactive lymph node cases were followed up for at least 12 months.
